# Fungi in the future: interannual variation and effects of atmospheric change on arbuscular mycorrhizal fungal communities

**DOI:** 10.1111/nph.13224

**Published:** 2015-01-05

**Authors:** T E Anne Cotton, Alastair H Fitter, R Michael Miller, Alex J Dumbrell, Thorunn Helgason

**Affiliations:** 1 Department of Biology, University of YorkYork, YO10 5DD, UK; 2 School of Biological Sciences, University of Essex, Wivenhoe ParkColchester, CO4 3SQ, UK; 3 Department of Animal and Plant Sciences, Alfred Denny Building, University of Sheffield, Western BankSheffield, S10 2TN, UK; 4 Biosciences Division, Argonne National Laboratory9700 S. Cass Avenue, Argonne, IL, 60439, USA

**Keywords:** 18S rRNA, arbuscular mycorrhizas, atmospheric change, free air concentration enrichment (FACE), Glomeromycota, microbial diversity, soil fungi, temporal dynamics

## Abstract

- Understanding the natural dynamics of arbuscular mycorrhizal (AM) fungi and their response to global environmental change is essential for the prediction of future plant growth and ecosystem functions. We investigated the long-term temporal dynamics and effect of elevated atmospheric carbon dioxide (CO_2_) and ozone (O_3_) concentrations on AM fungal communities.
- Molecular methods were used to characterize the AM fungal communities of soybean (*Glycine max*) grown under elevated and ambient atmospheric concentrations of both CO_2_ and O_3_ within a free air concentration enrichment experiment in three growing seasons over 5 yr.
- Elevated CO_2_ altered the community composition of AM fungi, increasing the ratio of Glomeraceae to Gigasporaceae. By contrast, no effect of elevated O_3_ on AM fungal communities was detected. However, the greatest compositional differences detected were between years, suggesting that, at least in the short term, large-scale interannual temporal dynamics are stronger mediators than atmospheric CO_2_ concentrations of AM fungal communities.
- We conclude that, although atmospheric change may significantly alter AM fungal communities, this effect may be masked by the influences of natural changes and successional patterns through time. We suggest that changes in carbon availability are important determinants of the community dynamics of AM fungi.

## Introduction

Since the industrial revolution, anthropogenic activities have caused an increase in carbon dioxide (CO_2_) levels from 280 to *c*. 400 ppm, and tropospheric ozone (O_3_) levels have more than doubled (Vingarzan, 2004; IPCC, 2007; Royal Society, 2008). The concentrations of both of these gases continue to rise, with CO_2_ concentrations predicted to reach 700 ppm by 2100 (Prather *et al*., 2003; IPCC, 2007; Cooper *et al*., 2010). The response of belowground organisms to these changes has received relatively little attention (Blankinship *et al*., 2011), hampering our ability to predict future ecosystem functioning (Singh *et al*., 2010). Therefore, it is important to better understand how global change will affect functionally significant components of soil biodiversity, for example, the arbuscular mycorrhizal (AM) belowground plant–microbe symbioses formed by fungi in the Phylum Glomeromycota (Johnson *et al*., 2013). These functionally important fungi are widely distributed, colonizing approximately two-thirds of plant species (Fitter & Moyersoen, 1996), and play central roles in many ecosystem processes. They have diverse functions, affecting plant growth, nutrient acquisition, drought and pathogen resistance, and heavy metal tolerance (reviewed in Smith & Read, 2008). Furthermore, functional differentiation among AM fungal taxa (Munkvold *et al*., 2004; Jansa *et al*., 2008) means that the composition and diversity of communities of these fungi can affect plant nutrient status (Jansa *et al*., 2008), community diversity (Blankinship *et al*., 2011) and plant productivity (Wagg *et al*., 2011).

It is likely that AM fungi will be affected by atmospheric change, as their sole carbon source is from their host plant roots (Smith & Read, 2008) and increases in CO_2_ and O_3_ increase and decrease plant photosynthesis and subsequent carbon allocation below ground, respectively (Andersen, 2003; Drigo *et al*., 2010). It has recently been suggested that a change in carbon availability is likely to influence AM fungal community composition and diversity by the same mechanisms by which nutrient availability influences plant communities (Dumbrell *et al*., 2011). Studies of plant communities have suggested that increasing the limiting resource which structures communities reduces evenness, as certain species often benefit disproportionately (Tilman, 1987; Bobbink *et al*., 1998; Stevens *et al*., 2004). In the same manner, in carbon-limited AM fungal communities, increased carbon availability under elevated CO_2_ (eCO_2_) would be predicted to decrease AM fungal community evenness, and elevated ozone (eO_3_) to increase it. However, although total soil fungal diversity has been shown to be related to resource availability (Waldrop *et al*., 2006), these hypotheses on the effects of global change on AM fungi have yet to be tested. The few studies that have examined whether atmospheric change will affect AM fungal community composition and diversity have been limited in their scope. For example, no previous studies have examined the effect of O_3_, and those examining CO_2_ have either used gas chambers or artificial laboratory microcosms (e.g. Treseder *et al*., 2003; Drigo *et al*., 2013), which may produce unnatural environmental growth conditions, or have characterized communities using spores (Wolf *et al*., 2003; Gamper *et al*., 2004), which only reflect fungal reproduction (Clapp *et al*., 1995).

Strong seasonal patterns of root growth are also significant drivers of variability in belowground carbon delivery. The first colonization of a plant in the growing season by a particular AM fungus is thought to influence community composition as a result of priority effects (Dumbrell *et al*., 2010a). In arable agriculture, where all root systems grow from seeds every year, inoculum potential and thus encounter rates between root and AM fungi at the beginning of the season are therefore likely to exert a strong influence on AM fungal community structure both within and between years. However, so far, there have been no in-depth studies of interannual variation in AM fungal communities in such systems.

In this study, we used molecular methods to characterize the AM fungal communities in roots of soybean (*Glycine max*) grown in the field and exposed to eO_3_ and eCO_2_ (predicted levels for 2050) from the Soybean Free Air Concentration Enrichment (SoyFACE) facility in Illinois, USA in three growing seasons over 5 yr. We predicted that eCO_2_ and eO_3_ would affect AM fungal community composition, and tested the following hypotheses: eCO_2_ would reduce the AM fungal community diversity by reducing evenness, and eO_3_ would increase diversity by increasing evenness. We also sought to measure interannual differences among the AM fungal communities.

## Materials and Methods

### Study site, atmospheric manipulation and plant sampling

Roots were sampled from the SoyFACE experiment (Illinois, USA; 40°03′21.3″N, 88°12′3.4″W; detailed descriptions in Moran & Jastrow, 2010). Briefly, the facility consisted of monocultures of *Glycine max* L., cv Pioneer 93B15, in which four experimental plots (called rings) of each of four different atmospheric conditions had been established since 2002, consisting of current and simulated levels of CO_2_ or O_3_ predicted for 2050: eCO_2_ (550 ppm) with ambient ozone (aO_3_); eO_3_ (1.2 times aO_3_ in 2004 and 2006 and 1.6 times aO_3_ in 2008) with ambient CO_2_ (aCO_2_); eCO_2_ and eO_3_; and an ambient control.

Atmospheric treatments were produced using a free air concentration enrichment (FACE) system, in which gases were released upwind from pipes surrounding 20 m diameter plots to maintain the desired concentrations. Soybeans were planted on 28 May 2004, 25 May 2006 and 17 June 2008, and were treated and grown using standard Midwestern agricultural practices, including growing in annual rotation with maize (*Zea mays*), and ploughed every year after the maize harvest. The eCO_2_ (but not eO_3_) fumigation treatments were continued through the maize rotation. When present, atmospheric treatment rings were located at the same positions within the fields every year.

Soybean plant roots were sampled from plants of approximately the same age from the 16 rings in each year on 29 July 2004 (62 days after planting, dap) 18 July 2006 (54 dap) and 14 August 2008 (58 dap). As a result of the biennial crop rotation and consistent location of treatment rings, plants were always sampled from the same locations in the same field across years. Eight, 10 and six plants were sampled from each ring in 2004, 2006 and 2008, respectively. Roots were washed with water to remove all surface soil. The resulting roots from 2004 and 2006 were freeze-dried and those from 2008 were dried at 60°C.

### Root processing and DNA extraction

Entire root systems were ground to powder with 2-mm-diameter tungsten carbide beads (Qiagen Ltd, Crawley, UK) using a ball mill (Retsch Mixer Mill, MM 301, Hann, Germany) at 23 Hz. Total community DNA was extracted from a subsample (*c*. 0.04 g) of the resulting homogenized powder. Roots from 2004 were pooled from plants originating in each ring before extraction, and DNA was separately extracted from individual plants in 2006 and 2008. All DNA extractions were performed using a PowerPlant DNA Isolation Kit (MoBio Laboratories Inc., Carlsbad, CA, USA) according to the manufacturer's instructions.

### Amplification of AM fungal DNA

Partial small subunit (SSU) ribosomal RNA gene fragments (*c*. 550 bp) were amplified from each DNA extract using HotstarTaq Plus DNA polymerase (Qiagen), with the universal eukaryotic primer NS31 (Simon *et al*., 1992) and primer AM1, which has enhanced specificity for AM fungi (Helgason *et al*., 1998). PCRs were carried out on 1 μl of template DNA extract in the presence of 0.64 mM deoxynucleoside triphosphates (dNTPs), 0.2 μM of each primer, 0.5 μg dried skimmed milk powder (Premier International Foods Ltd, Spalding, UK) to absorb inhibitors, and the manufacturer's reaction buffer in 25-μl reactions (PCR conditions: 95°C for 5 min; 40 cycles at 95°C for 30 s, 60°C for 30 s and 72°C for 15 s; and 72°C for 10 min on a Techne TC-512; Techne Co., Stone, UK). Samples that failed to amplify were reamplified using the same conditions, but with a 60-s elongation, or repurified using Centricon 10 microconcentrators (Amicon, Stonehouse, UK) before PCR.

### Clone library production, screening and sequencing

For an inventory of the AM fungal communities in the samples and to identify sequence types of particular interest, cloning and sequencing were performed. Equal volumes of successful amplifications were pooled to produce one amplification product for each ring and each year. These were cleaned using a QIAquick PCR Purification Kit (Qiagen) and products from the four rings of each atmospheric treatment for each year were pooled again. Each pooled sample was cloned into pGEM-T Easy vector (Promega Co., Madison, WI, USA) and transformed into *Escherichia coli* (DH5a; Invitrogen, Paisley, UK). Thus, one clone library was produced for each year and atmospheric treatment (4 treatments × 3 yr = 12 libraries). Putative positive transformants were screened using standard T7/SP6 PCR and restriction fragment length polymorphism (RFLP) patterns using the restriction enzymes HinfI, Hsp92-II and Rsa-I, and clones with unique RFLP patterns were sequenced using an ABI 3130 capillary sequencer (Applied Biosystems, Warrington, UK), as described in Dumbrell *et al*. (2010a). A further digestion using AluI or MboI (Promega), or additional sequencing, was performed if sequence analysis revealed that further differentiation of the initial RFLP types was required to confidently assign molecular operational taxonomic units (MOTUs). In total, 190 clones were sequenced, representing at least one and up to 12 clones of each RFLP type for each year and treatment in which it was found, depending on whether further digestions were required. Representative sequences of each MOTU were submitted to the EMBL Nucleotide Sequence Database (accession numbers KJ749677–KJ749694).

### Terminal restriction fragment length polymorphism (TRFLP) analysis

Each of the four rings for each atmospheric treatment at the SoyFACE experiment can be viewed as a true replicate. In order to utilize this level of replication, a higher throughput method for community analysis was required. The AM fungal communities of three of the plant root systems from each of the 16 treatment rings from 2006 and 2008 were therefore characterized using TRFLPs. Dual-labelled partial SSU rRNA gene sequences of AM fungi were produced as described previously for clone library production, but with the AM1 and NS31 primers labelled with FAM and HEX dyes, respectively (Eurofins MWG Operon, Ebersberg, Germany) and with an annealing temperature of 63°C. Labelled PCR products of *c*. 550 bp were cleaned by gel extraction using a 1.2% agarose gel and QIAquick Gel Extraction Kit (Qiagen) following the manufacturer's instructions.

Eight microlitres of each cleaned PCR product were separately digested using five units of HinfI and AluI (Promega) in a reaction volume of 15 μl containing the manufacturer's buffer and 2 μg BSA for 15 h, and restriction products were cleaned using a QIAquick PCR Purification Kit (Qiagen). The sizes and quantities of FAM-labelled AluI digests and HEX-labelled HinfI digests were determined against the GS600 LIZ size standard employing an ABI 3130 genetic analyser (and its supplied software: GeneMapper v4.0; Applied Biosystems).

### Data analysis

#### Sequence analysis

All sequences were compared with the National Center for Biotechnology Information (NCBI) database using BLAST (http://www.ncbi.nlm.nih.gov) and non-AM fungal sequences were removed from further analysis. The remaining sequences were aligned using MAFFT (http://mafft.cbrc.jp/alignment/server/) with default settings. Sequences were then assigned into MOTUs using a pairwise sequence similarity threshold of 97%. Fifty clones containing AM fungal SSU sequences were assigned to MOTUs from each of the 12 clone libraries using their RFLP patterns. One sequence from each MOTU was chosen at random and aligned with fungal sequences from three sources: reference sequences of identified AM fungi obtained from Krüger *et al*. (2012); their closest BLAST hits from the MaarjAM database, containing all known AM fungal 18S rRNA gene sequences divided into 288 ‘virtual taxa’ (VTX) based on their phylogenetic analysis (Öpik *et al*., 2010); and sequences of fungi whose speed of obtaining carbon from their host plants can be inferred from a previous study by Vandenkoornhuyse *et al*. (2007), in which AM fungi containing ^13^C, immediately after a pulse of ^13^CO_2_ was applied to their host plants, were identified. These sequences were then used for phylogenetic analysis. ClustalX (Thompson *et al*., 1997) was employed for sequence alignment and calculation of neighbour-joining phylogeny (Saitou & Nei, 1987) using *Paraglomus brasilianum* and *P. occultum* as outgroups, and a tree was drawn using Treeview (v1.6.6; Page, 1996). MOTUs were assigned to the VTX that the BLAST search showed they most closely matched under a sequence similarity threshold of ≥ 97%. Phylogenetic support was calculated using nonparametric bootstrapping with 10 000 pseudoreplicates (Felsenstein, 1985).

#### TRFLP analysis

FAM-labelled HinfI digests and HEX-labelled AluI fragments were not analysed, as *in silico* sequence analysis showed that they were uninformative (Supporting Information Table S1). Relative abundances of peaks greater than 100 fluorescent units in height and representing terminal restriction fragments (TRFs) longer than 100 bp were quantified using peak areas, a bin width of 2 bp and the local southern size calling method. Before further analysis, TRFs that represented on average < 5% of the TRFLP outputs of the samples in which they were present were excluded to eliminate noise, as in Dumbrell *et al*. (2010b). Thorough testing of the TRFLP protocol used has previously shown that it is highly repeatable with little bias, supporting the quantitative use of TRF abundance data from this experiment (Cotton *et al*., 2014).

#### Statistical analysis

To assess whether the AM fungal community from the roots analysed in this study had been thoroughly inventoried, a rarefaction curve was examined using combined data from all clone libraries employing Analytic Rarefaction 1.3 (available from http://strata.uga.edu/software/). Margalef's index (predominantly affected by species richness; *D*_Mg_; Magurran, 2004) for the AM fungal communities of individual plant root systems was calculated from the TRFLP data, using the percentage of each TRF for each enzyme digestion as the relative abundance of individuals of that type out of 100 individuals sampled, and the following formula:


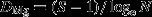
Eqn1

where *S* is the number of unique TRFs observed and *N* is the total percentage of TRFs observed across both enzymes (200). Simpson's index (affected by both richness and evenness; *D*; Magurran, 2004) was also calculated for the AM fungal communities of individual plant root systems, using TRFLP data as above and the following formula:


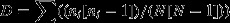
Eqn 2

where *N* is the total percentage of TRFs observed (200) and *n*_*i*_ is the proportion of TRFs of TRF *i* for each enzyme digestion. In all analyses and in the results presented, the reciprocal of Simpson's index (1/*D*) is used, so that an increasing value represents an increase in community evenness.

*In silico* analysis of the sequence alignment generated from the site showed that the only sequences of the Gigasporaceae found there (MOTU2 and MOTU3; Fig. S1) were predicted to produce similar sized TRFs. The only sequence of MOTU2 found was predicted to produce a 297-bp HEX-labelled HinfI TRF, and 29 of 30 sequences of MOTU3 were predicted to produce a similar 299-bp fragment. Such a small difference in size means that these two TRFs were empirically indistinguishable using the TRFLP protocol applied. The analysis of reference sequences of cultured, named AM fungi from all families also predicted this TRF to be produced by the majority of sequenced Gigasporaceae (Table S2). Moreover, the conserved nature of 18S rDNA sequences within the Gigasporaceae prevents the AM1 and NS31 amplicons from providing genus- or species-level resolution of the fungi in this family found at the SoyFACE site (Fig. S1). We therefore propose that the 297–299-bp TRF is considered as diagnostic for the family Gigasporaceae in this study. A previous experiment to empirically test for biases in the TRFLP method and the use of diagnostic TRFs at this experiment also supported the use of this fragment to estimate the relative abundance of this taxon (described in Cotton *et al*., 2014). The average relative abundance of Gigasporaceae in each plant in each of the treatment rings in both years was therefore calculated using the relative abundances of the 297–299-bp HEX-labelled HinfI TRF.

Detrended correspondence analysis (DCA) with downweighting of rare species was employed to characterize, analyse and visualize the overall community composition of individual plants using the TRFLP data. DCA was performed on the TRFLP data for each of the 96 plants analysed. The average DCA axis 1 and 2 scores were calculated for each of the 16 treatment rings in both years, and compared across treatments.

We used linear mixed effects models (LMMs) to examine the influence of CO_2_ and O_3_ treatments and year of sampling on the diversity (Margalef's and Simpson's indices) and composition (DCA axis 1 and 2 scores and percentage of Gigasporaceae) of AM fungal communities. In all cases, average values of each of these measures for plants in each of the 16 rings were used to avoid pseudoreplication. A square root transformation was applied to the Simpson's diversity index values and an arcsine square root transformation was applied to the relative abundances of Gigasporaceae before analysis to meet the assumptions of the models. We constructed two separate models to account for temporal (sampling year) and spatial (ring location) non-independence. First, we examined the main treatment effects of CO_2_ and O_3_, and the interactions between them, on AM fungal communities (diversity and composition), including these as fixed factors and sampling year and ring as random factors. We then examined year of sampling as a main treatment effect, including this within our fixed factors alongside any interaction effects with CO_2_ and O_3_ treatments, whilst keeping ring as a random factor. Models were fitted using maximum likelihood.

To confirm LMM analysis based on DCA axes, we also used Permutational Multivariate Analysis of Variance (PERMANOVA) to quantify the effect sizes of atmospheric treatments and year of sampling on AM fungal communities. A stepwise three-way PERMANOVA (permutations = 10 000) was performed on averaged TRFLP data from each ring, based on Bray–Curtis distances and using CO_2_ levels, O_3_ levels and year of sampling as factors, to examine the variation explained by each factor once all other variables had been accounted for.

All analyses were performed using the R statistical language (R Development Core Team, 2007) with PERMANOVA analysis and calculation of DCA scores conducted using the ‘vegan’ library (Oksanen *et al*., 2013), and LMMs were fitted using the nlme library (Pinheiro *et al*., 2014).

## Results

### AM fungal identities and sampling intensity

We identified 18 MOTUs from three families (Gigasporaceae, Glomeraceae and Acaulosporaceae) from the roots obtained from SoyFACE (Fig. S1). Visual inspection of the rarefied species accumulation curve showed that MOTU accumulation had approached an asymptote (Fig. S2), indicating that the community had been thoroughly inventoried.

### AM fungal community composition

**Fig 1:**
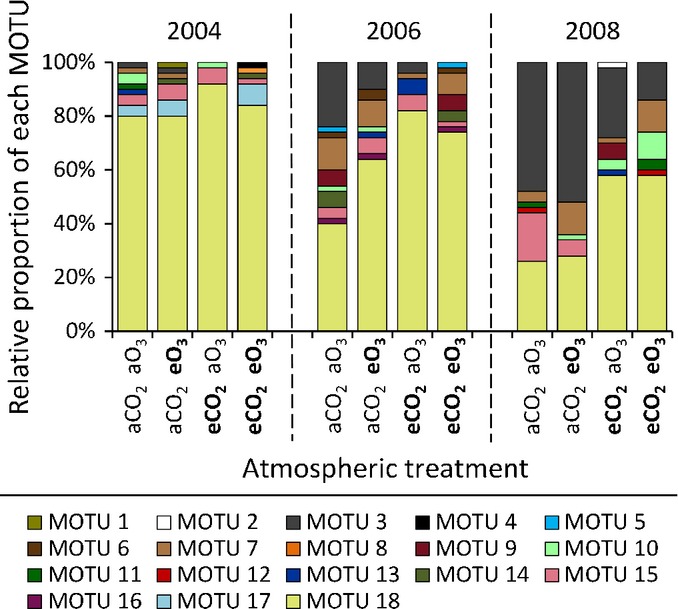
Stacked bar charts showing the proportion of sequences of each arbuscular mycorrhizal fungal molecular operational taxonomic unit (MOTU) in each year and atmospheric treatment (*n* = 50 for each community). MOTU numbers correspond to those used in Supporting Information Fig. S1. Atmospheric treatments are ambient carbon dioxide (aCO_2_), elevated carbon dioxide (eCO_2_), ambient ozone (aO_3_) and elevated ozone (eO_3_).

Of the 18 fungal taxa present, clone analysis showed that the relative abundance of MOTU3 (identifiable only to family level of Gigasporaceae because of the conserved nature of 18S rRNA genes within this group, and most similar to Öpik's VTX39) was consistently lower under eCO_2_ (mean = 7%) than under aCO_2_ (mean = 23%; Figs 1, S1). By contrast, the abundance of MOTU18 (putatively identified as *Rhizophagus irregularis* and most similar to Öpik's VTX114) was consistently higher under eCO_2_ (mean = 73%) than under aCO_2_ (mean = 53%; Figs 1, S1).

**Fig 2:**
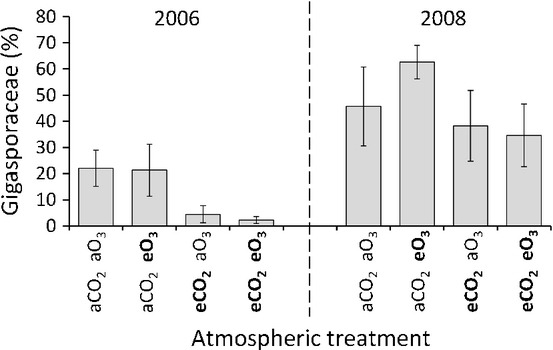
Relative abundance of Gigasporaceae in different atmospheric treatments and years as assessed using a 297–299-bp terminal restriction fragment (TRF) considered to be diagnostic for this taxon and terminal restriction fragment length polymorphism (TRFLP) analysis. Atmospheric treatments are ambient carbon dioxide (aCO_2_), elevated carbon dioxide (eCO_2_), ambient ozone (aO_3_) and elevated ozone (eO_3_). Error bars represent ± SE among replicate treatment rings (*n* = 4).

**Fig 3:**
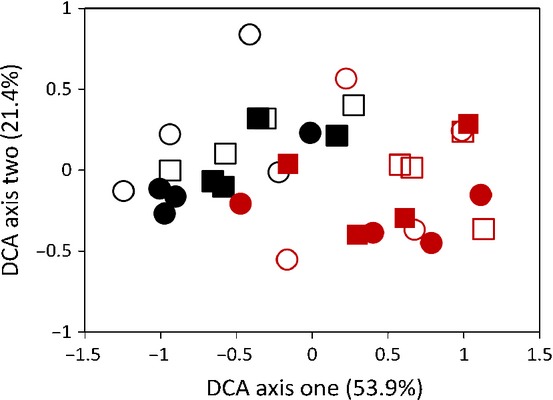
Detrended correspondence analysis (DCA) ordination plot of arbuscular mycorrhizal fungal data from terminal restriction fragment length polymorphism (TRFLP) analysis. Percentage values on the axes represent the variation in the fungal taxa abundance matrix explained by each axis. Different coloured symbols represent different years (black symbols, 2006; red symbols, 2008) and different shapes and shadings represent atmospheric treatments (circles, elevated CO_2_; squares, ambient CO_2_; open symbols, elevated O_3_; closed symbols, ambient O_3_).

Differences in community composition between CO_2_ treatments were also confirmed by TRFLP analysis by demonstrating that the relative abundance of the Gigasporaceae (measured using the 297–299-bp TRF) was lower under eCO_2_ than under aCO_2_ (*F*_1,12_ = 5.48, *P* = 0.037; Fig. 2). DCA of the TRFLP data explained 75% of the variation within the data across the first two ordination axes (Fig. 3). However, there was no significant main effect of CO_2_ treatment on the broad-scale composition of AM fungal communities (DCA axis 1: *F*_1,12_ = 1.07, *P* = 0.32; DCA axis 2: *F*_1,12_ = 0.67, *P* = 0.43; Fig. 3; PERMANOVA: *F*_1,28_ = 1.88, *R*^2^ = 0.035, *P* = 0.15). There was also no significant main effect of ozone treatment on the overall composition of AM fungal communities (DCA axis 1: *F*_1,12_ = 0.12, *P* = 0.74; ANOVA DCA axis 2: *F*_1,12_ = 3.06, *P* = 0.11; Fig. 3; PERMANOVA: *F*_1,28_ = 1.49, *R*^2^ = 0.028, *P* = 0.213) or the relative abundance of Gigasporaceae (*F*_1,12_ = 0.002, *P* = 0.97; Fig. 2).

Differences in community composition between years were found in the TRFLP data. The relative abundance of Gigasporaceae was significantly higher in 2008 than in 2006 (*F*_1,12_ = 27.21, *P* < 0.001; Fig. 3), and the year of sampling explained a significant amount of variation in DCA axis 1 scores, showing that there were also significant differences in overall community composition between years (*F*_1,12_ = 48.60, *P* < 0.001). This was confirmed by PERMANOVA, which also showed that the year of sampling had a significant effect on the AM fungal communities (*F*_1,28_ = 22.83, *R*^2^ = 0.421, *P* < 0.001). Importantly, PERMANOVA also showed that 42% of the variation in the TRFLP data was explained by the year of sampling, after accounting for the effects of CO_2_ and O_3_ treatments, compared with 3.5% by CO_2_ treatment and 2.8% by O_3_ treatment after accounting for the other factors.

There were no significant interactions between year, CO_2_ and O_3_ treatments on DCA axis 1 and axis 2 values or the percentage of Gigasporaceae (all *P* ≥ 0.39).

### AM fungal richness and evenness

**Fig 4:**
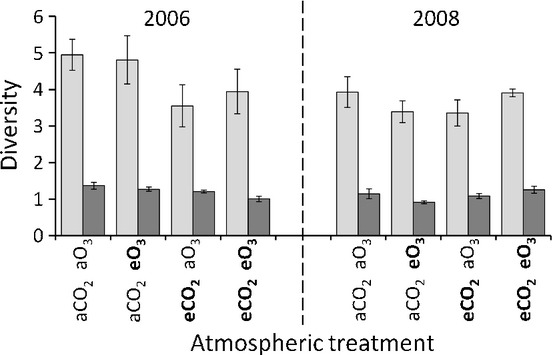
Arbuscular mycorrhizal fungal diversity (reciprocal Simpson's index, light grey bars) and richness (Margalef's index, dark grey bars) of communities from each atmospheric treatment and year determined using terminal restriction fragment length polymorphism (TRFLP) analysis. Atmospheric treatments are ambient carbon dioxide (aCO_2_), elevated carbon dioxide (eCO_2_), ambient ozone (aO_3_) and elevated ozone (eO_3_). Error bars represent ± SE of the means among replicate treatment rings (*n* = 4).

There were no significant main effects of sample year, CO_2_ and O_3_ levels on community richness (Fig. 4; Margalef's index: *F*_1,12_ = 3.97, *P* = 0.07; *F*_1,12_ = 0.24, *P* = 0.63; and *F*_1,12_ = 1.60, *P* = 0.23, respectively) or community evenness (Fig. 4; Simpson's index: *F*_1,12_ = 3.55, *P* = 0.08; *F*_1,12_ = 2.79, *P* = 0.12; and *F*_1,12_ = 0.04, *P* = 0.84, respectively) calculated from the TRFLP data. However, there was a significant year by CO_2_ level and year by CO_2_ by ozone level interaction for Margalef's index (*F*_1,12_ = 9.57, *P* = 0.01 and *F*_1,12_ = 5.14, *P* = 0.04, respectively). The magnitude of these differences was small, despite being significant (Fig. 4). For example, the year by CO_2_ interaction was caused by the average number of unique TRFs being one lower under eCO_2_ than under aCO_2_ in 2006, and one higher in 2008, creating a 16% decrease and 18% increase in Margalef's index, respectively (Fig. 4). All other interactions for Margalef's and Simpson's indices were not significant (*P* ≥ 0.12).

## Discussion

### eCO_2_ and AM fungal communities

This experiment demonstrates that eCO_2_ can alter *in planta* AM fungal community composition in the field. Using cloning and sequencing, we consistently observed lower relative abundance of the Gigasporaceae under eCO_2_ than under aCO_2_ across three growing seasons over 5 yr at the field level (Fig. 1). The same trend was also observed in both seasons examined at the individual plant level (Fig. 2). The use of replicate samples employing high-throughput TRFLP analysis allowed this to be statistically tested and revealed a significant reduction in the abundance of Gigasporaceae under eCO_2,_, although the variability was far higher in 2008 than in 2006 (Fig. 2). Furthermore, as almost all (>99.8%) of the other AM fungi at the site were Glomeraceae (Figs 1, S1), this represents a significant increase in the abundance of Glomeraceae relative to Gigasporaceae under eCO_2_ among treatments within years. This is consistent with other studies, despite disparate methodologies and timings. For example, the relative abundance of *Gigaspora* sp. extraradical mycelium was found to be reduced by an abrupt increase of CO_2_ to 550 ppm (Klironomos *et al*., 2005), and a study of the responses of different species of AM fungi grown in isolation to eCO_2_ also showed that it enhanced the intraradical growth of *Glomus intraradices* more than that of other fungal species (Klironomos *et al*., 1998). However, these studies were pot-based and therefore involved extremely artificial edaphic conditions and spatial constraints on root and fungal growth, and did not examine long-term, chronic effects of atmospheric treatments. By using a FACE experiment, we studied more realistic simulations of conditions which plants and AM fungi will experience in the future, thereby providing the first ever predictions of the effects of atmospheric change on *in planta* AM fungal field communities.

The results also show that a particular species of Glomeraceae (MOTU18; putatively identified as *Rhizophagus irregularis*) was consistently favoured under eCO_2_ (Figs 1, S1), although it should be noted that this could not be statistically tested as the laborious nature of clone library production and analysis required to examine this taxon prevented replication within years. This sequence is identical to a sequence type previously found to be effective at obtaining recently fixed photosynthate (Vandenkoornhuyse *et al*., 2007; accession no. EF041056, Fig. S1). In that study, ^13^CO_2_ pulse application and ^13^C-enriched RNA extraction from roots were used to determine the timing and identity of AM fungi obtaining recently fixed carbon from three host plants. The only AM fungal sequence always found to contain ^13^C immediately after labelling in all plants matched MOTU18 (Fig. S1), implying that it is a fungus particularly effective at quickly obtaining carbon from host plants. As previously stated, this increased relative abundance of Glomeraceae under eCO_2_ was further supported and found to be significant in the TRFLP data of AM fungal communities in individual plants. This is consistent with a stable isotope probing (SIP) study by Drigo *et al*. (2010), showing that, under eCO_2_, the Glomeraceae obtained the most recently fixed photosynthate. Moreover, a *Glomus* sp. was found to more competitively colonize plant roots with higher carbohydrate concentrations created by reducing soil phosphorus concentrations (Pearson *et al*., 1994). The consistency of findings between pot-based studies and this field study suggests that, despite their different environmental conditions, a common mechanism drives the changes. This, combined with the known properties of the fungal taxon favoured under eCO_2_, strongly suggests that changes in carbon allocation to roots caused the AM fungal community changes detected in this study.

The establishment of the mechanisms by which these alterations occur will be vital for predicting how global change will affect mycorrhizas and, consequently, ecosystem functioning. Currently, little is known about the mechanisms and regulation of resource exchange between plants and AM fungi. Fitter (2006) proposed that AM fungi create a localized increase in nutrient concentrations within the root, to which the plant then allocates more carbon, which is then available to the fungus. This mechanism suggests that the fungi that produce the greatest enhancement in host plant nutrient uptake under eCO_2_ will be best placed to benefit from increased carbon availability in this atmosphere, and will therefore increase in relative abundance.

This prediction is consistent with observations from this study, as the different fungi favoured under eCO_2_ and aCO_2_ have distinct life history strategies that are likely to produce different responses to additional carbon availability. Culturable *Glomus* species are thought to be r-strategists, growing and providing phosphorus to their host plants quickly (Boddington & Dodd, 1999; Sýkorová *et al*., 2007), whereas members of Gigasporaceae are K-strategists and grow slowly, accumulating hyphal phosphate before slowly releasing it to their host plants (Boddington & Dodd, 1999; de Souza *et al*., 2005). It is therefore likely that the members of Glomeraceae are more capable of using the extra carbon available under eCO_2_ to provide plants with more nutrients, consequently obtaining more carbon and increasing their relative abundance compared with the Gigasporaceae. The community changes detected in this study may therefore follow the principle of ‘optimal allocation’ (Johnson *et al*., 2013), whereby plants and fungi respond to global change by preferentially allocating resources towards increasing the supply of the most limiting resource, suggesting that the community changes detected could create a more efficient symbiosis.

Alternatively, from a mycocentric perspective, some fungi may be capable of obtaining extra carbon from host plants irrespective of their ability to enhance plant growth (Alberton *et al*., 2005). AM fungi obtain carbon using monosaccharide transporters (Helber *et al*., 2011), and it is possible that *Glomus* species have higher capacity hexose transporters than species of the Gigasporaceae, making them inherently better at quickly obtaining extra carbon allocated below ground under eCO_2_, even if they do not provide plants with extra nutrients. The fact that the fungus consistently favoured under eCO_2_ (MOTU18) is known to quickly obtain host plant carbon would be consistent with this hypothesis if the activity of transporters could be confirmed. We therefore suggest two alternative hypotheses to explain how AM fungi respond to eCO_2_, which can only be resolved through better understanding of AM fungal function. In particular, experiments are needed in which the ability of different fungi to provide nutrients to plants and carbon supplies to fungi are simultaneously manipulated. This could be achieved using a combination of root cultures, whereby carbon concentrations in host roots are directly manipulated, as used by Kiers *et al*. (2011), and plants are grown with individual fungal isolates and nutrient availabilities under different CO_2_ concentrations.

In addition to altering the efficiency of carbon and soil nutrient exchange, the detected changes in AM fungal community composition may also have significant impacts on other processes, as other functional traits are also conserved at the family level (Powell *et al*., 2009). For example, in general, members of the Glomeraceae have been shown to reduce pathogen infection more than those of Gigasporaceae (Sikes *et al*., 2010), but produce less extraradical mycelium (Maherali & Klironomos, 2007), which may have implications for soil stability (Leifheit *et al*., 2014). Further experiments should therefore focus on the functional changes associated with the predicted AM fungal community shifts. These studies should incorporate experiments in which the atmospheric CO_2_ concentration is gradually increased, as it has been found previously that AM fungal community responses to long-term CO_2_ changes can be more subtle than the responses to abrupt increases, such as those applied at SoyFACE (Klironomos *et al*., 2005), implying that the changes that we detected may be greater than those that will actually occur in response to global change.

Despite the observation that eCO_2_ alters the community composition and favours particular fungi, it did not reduce the evenness of the communities (Fig. 4). This could be because MOTU18 was not favoured sufficiently strongly under eCO_2_ to dominate the community and significantly reduce community evenness. We can speculate that crop rotation at the site and hence the change in host plant identity every year, a form of disturbance that would not occur in more natural systems, could be driving this. Alternatively, it could be that the analysis methods used were not sufficiently sensitive to detect any changes. Higher resolution community characterization, for example using next-generation sequencing, as employed in Öpik *et al*. (2010) and Dumbrell *et al*. (2011), would resolve this issue.

### eO_3_ and AM fungal communities

No effects of eO_3_ on AM fungal community composition (Figs 3), richness and evenness (Fig. 4) were observed. This could be because the effects of ozone on plant photosynthesis are cumulative (Morgan *et al*., 2004) and the plants sampled were young (54–62 dap), and so their exposure to eO_3_ was therefore limited and thus may have been insufficient to alter plant growth and thereby affect AM fungi in this experiment. This is the first investigation to examine whether ozone affects AM fungal communities, but the proposed explanation for the lack of observed effects is supported by other studies of AM fungal growth and microbial communities. For example, *Glomus fasciculatum* colonization levels were unaffected by ozone after 6 wk, but significantly reduced after 9 wk (McCool & Menge, 1984), and microbial communities of meadow mesocosms were unaffected by eO_3_ after 2 months, but significantly altered after 2 yr (Kanerva *et al*., 2008). The AM fungal communities of older plants are therefore more likely to be affected by ozone, and the results of this study should not be extrapolated to suggest that ozone cannot influence AM fungal communities.

### Interannual variation in AM fungal communities

In addition to investigating atmospheric change, this experiment is one of the most extensive studies examining the long-term temporal variation in AM fungal communities. We observed significant differences in the overall composition of AM fungi in the same field between different years (Figs 3). In particular, the average relative abundance of the Gigasporaceae was over three times higher in 2008 than in 2006 (Fig. 2). Indeed, the differences between years were far greater than those between CO_2_ treatments (Figs 3), and the year of sampling affected broad-scale community composition, whereas none of the atmospheric treatments had the same effect (Fig. 3). PERMANOVA showed that CO_2_ levels explained 3.5% of the variability in the data, compared with 42% explained by the year of sampling. The relative abundance of the Gigasporaceae also differed far more between years than between CO_2_ treatments (Fig. 2), suggesting that the effect of atmospheric change may be masked by the natural dynamics of AM fungal populations. It should be noted that, in this study, different sample preservation methods were used between years, as samples from 2006 were freeze-dried and those from 2008 were oven dried. Although we are unaware of any evidence that these two methods differentially preserved DNA from different fungal taxa, as the effects of DNA preparation methods have focused on roots colonized by a single AM fungus (Bainard *et al*., 2010) the possibility that this contributed to the observed interannual differences cannot be dismissed. Nonetheless, this study provides strong evidence that there can be large interannual differences in AM fungal intraradical communities of plants of the same age. This is a particularly novel finding as previous molecular studies that have sampled the same ecosystem in successive years have either focused on variation within growing seasons (Daniell *et al*., 2001) or sampled the same plant cohort through time, and were unable to differentiate between the differences caused by increased plant age and sampling time (Husband *et al*., 2002). Moreover, this experiment also highlights the urgent need for long-term monitoring of microbial communities to understand the mechanisms that shape them. Without this knowledge, the manipulation of soil microbial communities to enhance crop growth and to predict responses to global change will be challenging.

### Conclusions

Overall, this study represents the most detailed examination to date of long-term patterns of AM fungal communities *in planta* and how they are affected by atmospheric change, providing unparalleled insights into how these important soil microbes will change in the future. Critically, we have demonstrated that, in this disturbed agricultural system, the AM fungal community changes dramatically between years, suggesting that studies carried out during a single year may be difficult to interpret. In doing so, it also highlights the need for more information on the mechanisms that shape AM fungal communities, how global change will affect soil microbes and the functional consequences of these changes.

## Supporting Information

**Fig. S1** Neighbour-joining phylogenetic tree of the molecular operational taxonomic units of arbuscular mycorrhizal fungal taxa from the Soybean Free Air Concentration Enrichment experiment. **Fig. S2** Rarefaction curve for clone library analysis showing the relationship between sampling effort and arbuscular mycorrhizal fungal richness.

**Table S1** Predicted terminal restriction fragment lengths of arbuscular mycorrhizal fungal sequences obtained from the Soybean Free Air Concentration Enrichment experiment **Table S2** Predicted terminal restriction fragment lengths of arbuscular mycorrhizal fungal sequences of different species from different families generated using an 18S nuclear small subunit (SSU) rRNA gene alignment by Krüger *et al*. (2012)

## References

[b1] Alberton O, Kuyper TW, Gorissen A (2005). Taking mycocentrism seriously: mycorrhizal fungal and plant responses to elevated CO_2_. New Phytologist.

[b2] Andersen CP (2003). Source–sink balance and carbon allocation below ground in plants exposed to ozone. New Phytologist.

[b3] Bainard LD, Klironomos JN, Hart MM (2010). Differential effect of sample preservation methods on plant and arbuscular mycorrhizal fungal DNA. Journal of Microbiological Methods.

[b4] Blankinship JC, Niklaus PA, Hungate BA (2011). A meta-analysis of responses of soil biota to global change. Oecologia.

[b5] Bobbink R, Hornung M, Roelofs JGM (1998). The effects of air-borne nitrogen pollutants on species diversity in natural and semi-natural European vegetation. Journal of Ecology.

[b6] Boddington CL, Dodd JC (1999). Evidence that differences in phosphate metabolism in mycorrhizas formed by species of *Glomus* and *Gigaspora* might be related to their life-cycle strategies. New Phytologist.

[b7] Clapp JP, Young JPW, Merryweather JW, Fitter AH (1995). Diversity of fungal symbionts in arbuscular mycorrhizas from a natural community. New Phytologist.

[b8] Cooper OR, Parrish DD, Stohl A, Trainer M, Nédélec P, Thouret V, Cammas JP, Oltmans SJ, Johnson BJ, Tarasick D (2010). Increasing springtime ozone mixing ratios in the free troposphere over western North America. Nature.

[b9] Cotton TEA, Dumbrell AJ, Helgason T (2014). What goes in must come out: testing for biases in molecular analysis of arbuscular mycorrhizal fungal communities. PLoS ONE.

[b10] Daniell TJ, Husband R, Fitter AH, Young JPW (2001). Molecular diversity of arbuscular mycorrhizal fungi colonising arable crops. FEMS Microbiology Ecology.

[b11] Drigo B, Kowalchuk GA, Knapp BA, Pijl AS, Boschker HTS, van Veen JA (2013). Impacts of 3 years of elevated atmospheric CO_2_ on rhizosphere carbon flow and microbial community dynamics. Global Change Biology.

[b12] Drigo B, Pijl AS, Duyts H, Kielak AM, Gamper HA, Houtekamer MJ, Boschker HTS, Bodelier PLE, Whiteley AS, van Veen JA (2010). Shifting carbon flow from roots into associated microbial communities in response to elevated atmospheric CO_2_. Proceedings of the National Academy of Sciences, USA.

[b13] Dumbrell AJ, Ashton PD, Aziz N, Feng G, Nelson M, Dytham C, Fitter AH, Helgason T (2011). Distinct seasonal assemblages of arbuscular mycorrhizal fungi revealed by massively parallel pyrosequencing. New Phytologist.

[b14] Dumbrell AJ, Nelson M, Helgason T, Dytham C, Fitter AH (2010a). Idiosyncrasy and overdominance in the structure of natural communities of arbuscular mycorrhizal fungi: is there a role for stochastic processes?. Journal of Ecology.

[b15] Dumbrell AJ, Nelson M, Helgason T, Dytham C, Fitter AH (2010b). Relative roles of niche and neutral processes in structuring a soil microbial community. ISME Journal.

[b16] Felsenstein J (1985). Confidence-limits on phylogenies: an approach using the bootstrap. Evolution.

[b17] Fitter AH (2006). What is the link between carbon and phosphorus fluxes in arbuscular mycorrhizas? A null hypothesis for symbiotic function. New Phytologist.

[b18] Fitter AH, Moyersoen B (1996). Evolutionary trends in root–microbe symbioses. Philosophical Transactions of the Royal Society B.

[b19] Gamper H, Peter M, Jansa J, Lüscher A, Hartwig UA, Leuchtmann A (2004). Arbuscular mycorrhizal fungi benefit from 7 years of free air CO_2_ enrichment in well-fertilized grass and legume monocultures. Global Change Biology.

[b20] Helber N, Wippel K, Sauer N, Schaarschmidt S, Hause B, Requena N (2011). A versatile monosaccharide transporter that operates in the arbuscular mycorrhizal fungus *Glomus* sp is crucial for the symbiotic relationship with plants. Plant Cell.

[b21] Helgason T, Daniell TJ, Husband R, Fitter AH, Young JPW (1998). Ploughing up the wood-wide web?. Nature.

[b22] Husband R, Herre EA, Young JPW (2002). Temporal variation in the arbuscular mycorrhizal communities colonising seedlings in a tropical forest. FEMS Microbiology Ecology.

[b23] Solomon S, Qin D, Manning M, Chen Z, Marquis M, Averyt KB, Tignor M, Miller HL, IPCC (2007). Climate change 2007: the physical science basis. Contribution of Working Group I to the fourth assessment report of the Intergovernmental Panel on Climate Change.

[b24] Jansa J, Smith FA, Smith SE (2008). Are there benefits of simultaneous root colonization by different arbuscular mycorrhizal fungi?. New Phytologist.

[b25] Johnson NC, Angelard C, Sanders IR, Kiers ET (2013). Predicting community and ecosystem outcomes of mycorrhizal responses to global change. Ecology Letters.

[b26] Kanerva T, Palojärvi A, Rämöa K, Manninen S (2008). Changes in soil microbial community structure under elevated tropospheric O_3_ and CO_2_. Soil Biology and Biochemistry.

[b27] Kiers ET, Duhamel M, Beesetty Y, Mensah JA, Franken O, Verbruggen E, Felbaum CR, Kowalchuk GA, Hart MM, Bago A (2011). Reciprocal rewards stabilize cooperation in the mycorrhizal symbiosis. Science.

[b28] Klironomos JN, Allen MF, Rillig MC, Piotrowski J, Makvandi-Nejad S, Wolfe BE, Powell JR (2005). Abrupt rise in atmospheric CO_2_ overestimates community response in a model plant–soil system. Nature.

[b29] Klironomos JN, Ursic M, Rillig MC, Allen MF (1998). Interspecific differences in the response of arbuscular mycorrhizal fungi to *Artemisia tridentata* grown under elevated atmospheric CO_2_. New Phytologist.

[b30] Krüger M, Krüger C, Walker C, Stockinger H, Schüßler A (2012). Phylogenetic reference data for systematics and phylotaxonomy of arbuscular mycorrhizal fungi from phylum to species level. New Phytologist.

[b31] Leifheit EF, Veresoglou SD, Lehmann A, Morris EK, Rillig MC (2014). Multiple factors influence the role of arbuscular mycorrhizal fungi in soil aggregation – a meta-analysis. Plant and Soil.

[b33] Magurran AE (2004). Measuring biological diversity.

[b34] Maherali H, Klironomos JN (2007). Influence of phylogeny on fungal community assembly and ecosystem functioning. Science.

[b35] McCool PM, Menge JA (1984). Interaction of ozone and mycorrhizal fungi on tomato as influenced by fungal species and host variety. Soil Biology and Biochemistry.

[b36] Moran KK, Jastrow JD (2010). Elevated carbon dioxide does not offset loss of soil carbon from a corn–soybean agroecosystem. Environmental Pollution.

[b37] Morgan PB, Bernacchi CJ, Ort DR, Long SP (2004). An *in vivo* analysis of the effect of season-long open-air elevation of ozone to anticipated 2050 levels on photosynthesis in soybean. Plant Physiology.

[b38] Munkvold L, Kjøller R, Vestberg M, Rosendahl S, Jakobsen I (2004). High functional diversity within species of arbuscular mycorrhizal fungi. New Phytologist.

[b39] Oksanen J, Blanchet FG, Kindt R, Legendre P, Minchin PR, O'Hara RB, Simpson GL, Solymos P, Stevens MHH, Wagner H (2013). http://CRAN.R-project.org/package=vegan.

[b40] Öpik M, Vanatoa A, Vanatoa E, Moora M, Davison J, Kalwij JM, Reier U, Zobel M (2010). The online database MaarjAM reveals global and ecosystemic distribution patterns in arbuscular mycorrhizal fungi (Glomeromycota). New Phytologist.

[b41] Page RDM (1996). TreeView: an application to display phylogenetic trees on personal computers. Computer Applications in the Biosciences.

[b42] Pearson JN, Abbott LK, Jasper DA (1994). Phosphorus, soluble carbohydrates and the competition between two arbuscular mycorrhizal fungi colonising subterranean clover. New Phytologist.

[b43] Pinheiro J, Bates D, DebRoy S, Sarkar D, R Core Team (2014). http://cran.r-project.org/web/packages/nlme/index.html.

[b44] Powell JR, Parrent JL, Hart MM, Klironomos JN, Rillig MC, Maherali H (2009). Phylogenetic trait conservatism and the evolution of functional trade-offs in arbuscular mycorrhizal fungi. Proceedings of the Royal Society B.

[b45] Prather M, Gauss M, Berntsen T, Isaksen I, Sundet J, Bey I, Brasseur G, Dentener F, Derwent R, Stevenson D (2003). Fresh air in the 21st century?. Geophysical Research Letters.

[b46] Royal Society (2008). Ground-level ozone in the 21st century: future trends, impacts and policy implications. RS science policy report 15/08.

[b47] Saitou N, Nei M (1987). The neighbor-joining method: a new method for reconstructing phylogenetic trees. Molecular Biology and Evolution.

[b48] Sikes B, Powell JR, Rillig MC (2010). Deciphering the relative contributions of multiple functions within plant–microbe symbioses. Ecology.

[b49] Simon L, Lalonde M, Bruns TD (1992). Specific amplification of 18S fungal ribosomal genes from vesicular–arbuscular endomycorrhizal fungi colonizing roots. Applied and Environmental Microbiology.

[b50] Singh BK, Bardgett RD, Smith P, Reay DS (2010). Microorganisms and climate change: terrestrial feedbacks and mitigation options. Nature Reviews Microbiology.

[b51] Smith SE, Read DJ (2008). Mycorrhizal symbiosis.

[b54] de Souza FA, Dalpé Y, Declerck S, de la Providencia IE, Séjalon-Delmas N, Declerck S, Strullu DG, Fortin JA (2005). Life history strategies in Gigasporaceae: insight from monoxenic culture. In vitro culture of mycorrhizas.

[b55] Stevens CJ, Dise NB, Mountford JO, Gowing DJ (2004). Impact of nitrogen deposition on the species richness of grasslands. Science.

[b56] Sýkorová Z, Ineichen K, Wiemken A, Redecker D (2007). The cultivation bias: different communities of arbuscular mycorrhizal fungi detected in roots from the field, from bait plants transplanted to the field, and from a greenhouse trap experiment. Mycorrhiza.

[b57] Thompson JD, Gibson TJ, Plewniak F, Jeanmougin F, Higgins DG (1997). The CLUSTAL_X windows interface: flexible strategies for multiple sequence alignment aided by quality analysis tools. Nucleic Acids Research.

[b58] Tilman D (1987). Secondary succession and the pattern of plant dominance along experimental nitrogen gradients. Ecological Monographs.

[b59] Treseder KK, Egerton-Warburton LM, Allen MF, Cheng Y, Oechel WC (2003). Alteration of soil carbon pools and communities of mycorrhizal fungi in chaparral exposed to elevated carbon dioxide. Ecosystems.

[b60] Vandenkoornhuyse P, Mahé S, Ineson P, Staddon P, Ostle N, Cliquet JB, Francez AJ, Fitter AH, Young JPW (2007). Active root-inhabiting microbes identified by rapid incorporation of plant-derived carbon into RNA. Proceedings of the National Academy of Sciences, USA.

[b61] Vingarzan R (2004). A review of surface ozone background levels and trends. Atmospheric Environment.

[b62] Wagg C, Jansa J, Stadler M, Schmid B, van der Heijden MGA (2011). Mycorrhizal fungal identity and diversity relaxes plant–plant competition. Ecology.

[b63] Waldrop MP, Zak DR, Blackwood CB, Curtis CD, Tilman D (2006). Resource availability controls fungal diversity across a plant diversity gradient. Ecology Letters.

[b64] Wolf J, Johnson NC, Rowland DL, Reich PB (2003). Elevated CO_2_ and plant species richness impact arbuscular mycorrhizal fungal spore communities. New Phytologist.

