# Supplementary material for: Fungi in the future: interannual variation and effects of atmospheric change on arbuscular mycorrhizal fungal communities
Source: New Phytol. 2015 Jan 5;205(4):1598–607. doi: 10.1111/nph.13224 (PMC4338757; doi:10.1111/nph.13224)
Supplement: Supplementary file 1 [file nph0205-1598-sd1.pdf]

Supporting Information Figs S1 & S2

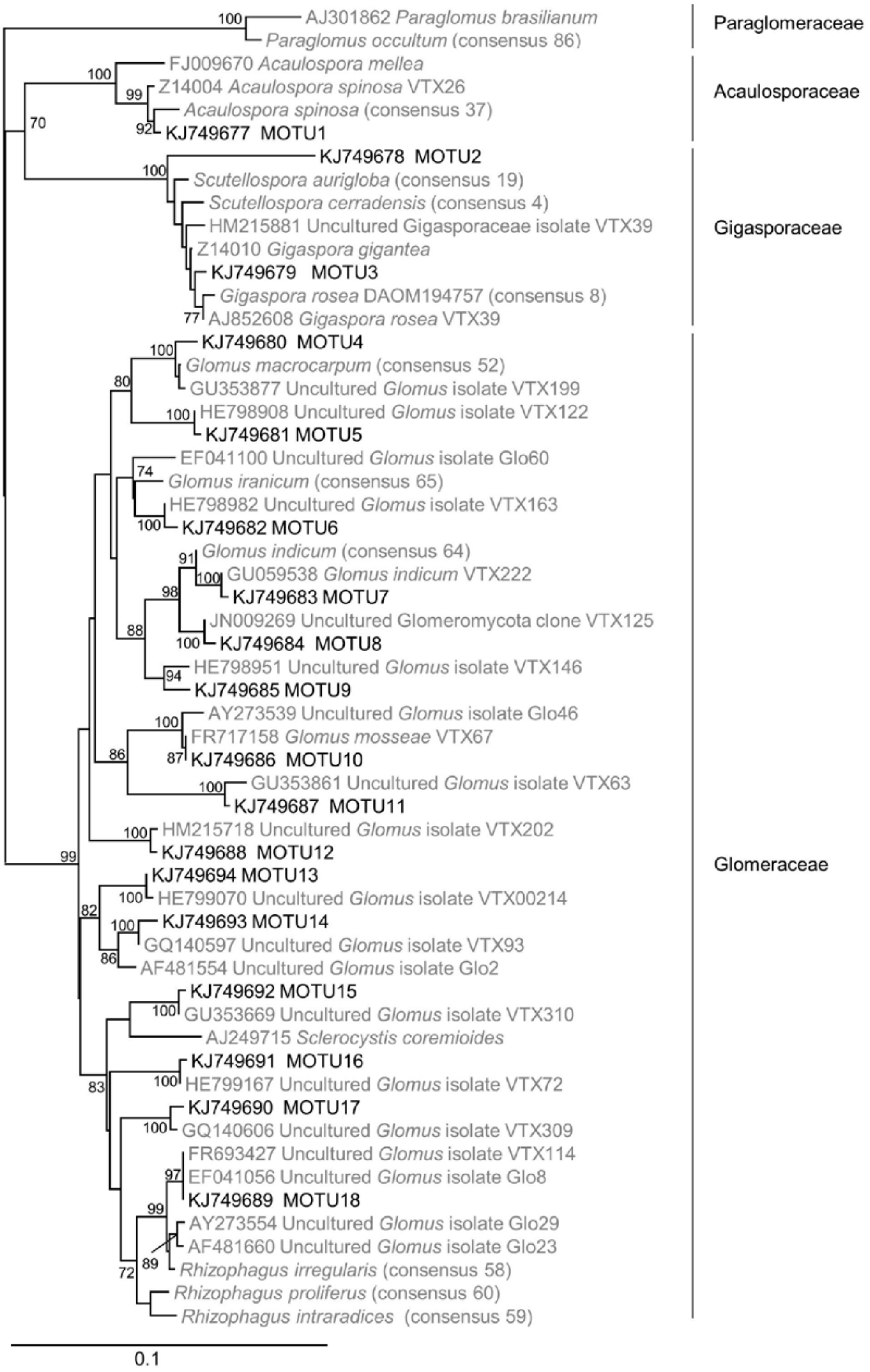

Fig. S1 Neighbour-joining phylogenetic tree showing the molecular operational taxonomic units (MOTUs) of arbuscular mycorrhizal (AM) fungal taxa from the Soybean free air concentration enrichment (SoyFACE) experiment. MOTUs identify sequences with >3% dissimilarity. Named reference sequences from Krüger *et al.* (2012), sequences from Vandenkoornhuyse *et al.* (2007) and the closest sequences in MaarjAM database including

their virtual taxa (VTX) numbers as defined by Öpik *et al.* (2010) are displayed, with accession numbers in grey. Bootstrap values ≥ 70% (10,000 replicates) are shown by the nodes to which they correspond. The scale bar represents 0.1 substitutions per nucleotide site. All sequences given from this experiment have been submitted to the EMBL Nucleotide Sequence Database (accession numbers KJ749677–KJ749694). Please note the similarities between MOTU3 and different genera of the Gigasporaceae caused by sequence conservation of the 18S rDNA gene within this family.

References

Krüger M, Krüger C, Walker C, Stockinger H, Schüßler A. 2012. Phylogenetic reference data for systematics and phylotaxonomy of arbuscular mycorrhizal fungi from phylum to species level. *New Phytologist* **193:** 970–984.

Öpik M, Vanatoa A, Vanatoa E, Moora M, Davison J, Kalwij JM, Reier U, Zobel M. 2010. The online database MaarjAM reveals global and ecosystemic distribution patterns in arbuscular mycorrhizal fungi (Glomeromycota). *New Phytologist* **188:** 223–241.

Vandenkoornhuyse P, Mahé S, Ineson P, Staddon P, Ostle N, Cliquet JB, Francez AJ, Fitter AH, Young JPW. 2007. Active root-inhabiting microbes identified by rapid incorporation of plant-derived carbon into RNA. *Proceedings of the National Academy of Sciences, USA* **104:** 16970–16975.

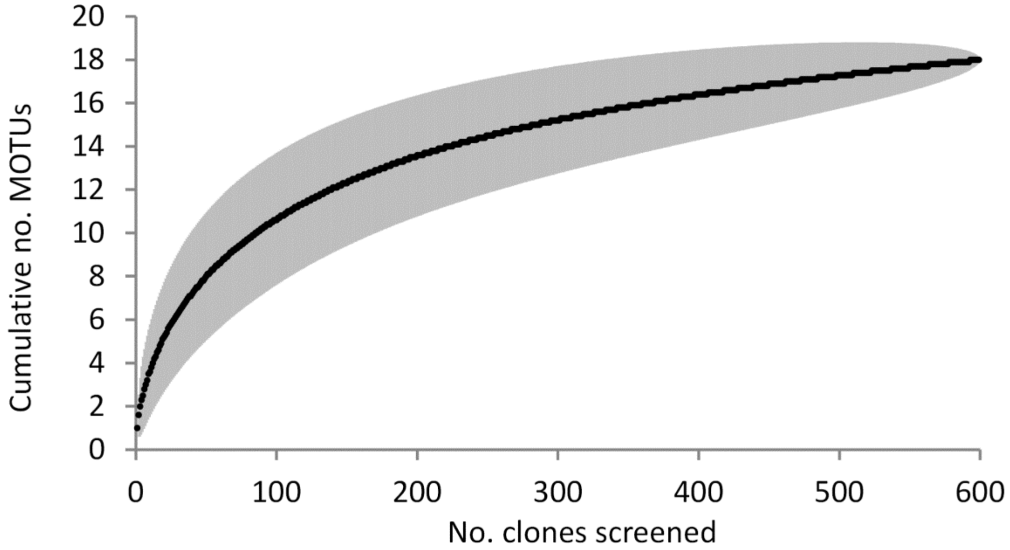

Fig. S2 Rarefaction curve for clone library analysis showing the relationship between sampling effort (number of clones screened) and arbuscular mycorrhizal fungal richness (the numbers of unique molecular operational taxonomic units, MOTUs) for data pooled from all 12 clone libraries (no. of sequences = 600). The grey shaded area represents the 95% confidence interval for the curve.
